# Global phylogeography suggests extensive eucosmopolitanism in Mesopelagic Fishes (*Maurolicus*: Sternoptychidae)

**DOI:** 10.1038/s41598-020-77528-7

**Published:** 2020-11-25

**Authors:** David J. Rees, Jan Y. Poulsen, Tracey T. Sutton, Paulo A. S. Costa, Mauricio F. Landaeta

**Affiliations:** 1 Department of Biological Sciences, University of Bergen, Thormøhlensgate 53A, 5006 Bergen, Norway; 2 Fish Section, Australian Museum, 6 College Street, Sydney, NSW 2010 Australia; 3 Department of Marine and Environmental Sciences, Nova Southeastern University, Dania Beach, FL 33004 USA; 4 Laboratório de Dinâmica de Populações Marinhas, Universidade Federal do Estado do Rio de Janeiro (UNIRIO), Rio de Janeiro, RJ 22290-240 Brazil; 5 Centro de Observación Marina para estudios de riesgos del ambiente costero (COSTA-R)tad de Ciencias del Mar y de Recursos Naturales, Universidad de Valparaíso, Avenida Borgoño, Reñaca, 16344 Viña del Mar, Chile

**Keywords:** Phylogenetics, Speciation, Marine biology

## Abstract

Fishes in the mesopelagic zone (200–1000 m) have recently been highlighted for potential exploitation. Here we assess global phylogeography in *Maurolicus*, the Pearlsides, an ecologically important group. We obtained new sequences from mitochondrial COI and nuclear ITS-2 from multiple locations worldwide, representing 10 described species plus an unknown central South Pacific taxon. Phylogenetic analyses identified five geographically distinct groupings, three of which comprise multiple described species. Species delimitation analyses suggest these may represent four species. *Maurolicus muelleri* and *M. australis* are potentially a single species, although as no shared haplotypes are found between the two disjunct groups, we suggest maintenance of these as two species. *Maurolicus australis* is a predominantly southern hemisphere species found in the Pacific, Indian and southern South Atlantic Oceans, comprising five previously allopatric species. *M. muelleri* (previously two species) is distributed in the North Atlantic and Mediterranean Sea. *Maurolicus weitzmani* (previously two species) inhabits the eastern equatorial Atlantic, Gulf of Mexico and western North and South Atlantic. *Maurolicus mucronatus* is restricted to the Red Sea. No *Maurolicus* have previously been reported in the central South Pacific but we have identified a distinct lineage from this region, which forms a sister group to *Maurolicus* from the Red Sea.

## Introduction

Mesopelagic fishes have been highlighted as a potential target for exploitation by fisheries and the biomarine industry in recent years, following revised biomass estimates of 10 billion metric tons^1,2^. While there is still uncertainty concerning the exact biomass involved, these estimates support the claim that mesopelagic fishes are the most abundant vertebrates on Earth^3^. Many mesopelagic fishes, typically present in the open ocean at depths between 200 and 1000 m during daytime, undergo daily vertical migrations in the water column. During these vertical migrations, they feed at shallower depths at night and retreat to deeper waters during the daytime, where they excrete, with important implications for oceanic biogeochemical cycles through trophic connectivity and organic carbon transport^4^. However, there remains a significant knowledge gap surrounding the distribution and composition of mesopelagic biodiversity and the potential impacts of harvesting these fishes are uncertain on scales that range from local mesopelagic communities to global processes.

Pearlsides of the genus *Maurolicus* (Stomiiformes: Sternoptychidae) have a circumglobal distribution, being found in all non-polar oceans, where they maintain enormous population sizes^5,6^. *Maurolicus* species are distributed throughout open oceans, along continental shelves and slopes, around isolated seamounts, and in some cases in fjord systems and inner seas^7,8^. Highly variable life-history parameters have been reported on a range of geographic scales^7,9,10^ and this plasticity has been suggested as a factor in the proliferation of *Maurolicus* worldwide^11^. This group has a complex taxonomic history, due in part to lack of variation in photophores used as distinguishing characters in other mesopelagic fishes. Six species were described by the early twentieth century, but lack of clear morphological separation led Grey^5^ to consider these as a single valid species, *Maurolicus muelleri* (Gmelin, 1789). A later revision of the genus by Parin and Kobyliansky^12^ resurrected the earlier species and proposed a number of new taxa, resulting in 15 species worldwide. However, many of these species remained poorly defined morphologically, with overlapping ranges of meristic and morphometric characters^13^. Since the 1996 revision, with the assumption of restricted species ranges, species diagnoses are often based on location of capture (e.g.^14^.).

Despite a large body of scientific work devoted to the distribution, life-history and ecology of *Maurolicus* from various regions, only a very limited number of studies have applied molecular data to species-level studies^13,15–18^. Initial examination of 16S rRNA data from *M. muelleri* (eastern North Atlantic Ocean) and *M. walvisensis* (eastern South Atlantic, off southern Africa) indicated a shallow divergence but Suneetha et al.^15^ concluded that these were two recently diverged species. Kim et al.^16^ expanded this work to include a third species, *M. japonicus* that, according to Parin and Kobyliansky^12^, is distributed in the western North Pacific around Japan and also an isolated population around Hawaii. This study maintained a distinct *M. muelleri* but found *M. japonicus* and *M. walvisensis* to be indistinguishable based on 16S and morphometric data. This treatment of *M. japonicus* and *M. walvisensis* as conspecifics was subsequently adopted by Habib et al.^17^, who referred to these as the Korean and Namibian populations of *M. japonicus*.

A multi-gene study of *Maurolicus* from across a wider geographical range was carried out by Rees et al.^13^ and also incorporated morphological data in a broader attempt to assess species status. As with the earlier studies, this work utilised 16S data but also included more variable COI data and nuclear gene sequences (ITS-2). From five putative species, this study identified three clear groupings with general congruence between morphological and molecular analyses, but which conflicted with previously recognised species. A shallow genetic divergence was observed between a ‘northern’ clade comprising North Atlantic *M. muelleri* and *M. amethystinopunctatus* (Mediterranean and eastern North Atlantic around the Azores) and a ‘southern’ clade consisting of *M. australis* (New Zealand and southern Australia) and *M. walvisensis* (Namibia and South Africa); by extension this would also include *M. japonicus*^16^, subsequently verified by Terada et al.^18^. A third clade comprised *M. weitzmani* from the eastern equatorial Atlantic and the western North Atlantic. While *M. weitzmani* was distinct from other *Maurolicus* on a molecular and morphological basis, analyses indicated a potential need to synonymise taxa in both the northern and southern clades.

The work presented here is a significant expansion of earlier work on *Maurolicus* and incorporates new data for taxa from Chile (*M. parvipinnis*), the Galápagos Islands (*M. breviculus*), Brazil, (*M. stehmanni*), the Red Sea (*M. mucronatus*), Japan (*M. japonicus*) and an unknown *Maurolicus* species from Fiji, French Polynesia and American Samoa, the first known records of any *Maurolicus* from this region. We also expand our earlier sampling of *M. weitzmani* (Gulf of Mexico) and *M. walvisensis* (Southwest Indian Ridge). With the addition of this material we are now able to assess genetic patterns among *Maurolicus* on a global scale, with ten of the 15 described species represented. In a previous study^13^ we demonstrated that morphological characters used in *Maurolicus* species descriptions by Parin and Kobyliansky^12^ are, in many cases, inadequate for reliable species determination. Our aims in the present work are to shed further light on genetic variation within the genus, with a view to establishing a reliable ‘molecular backbone’ against which to reassess species diversity, distribution and the evolutionary history of this important component of pelagic ecosystems. We also apply four commonly used species delimitation methods to objectively view the observed genetic variation and to provide a clear basis for further hypothesis building and testing.

## Materials and methods

### Sampling

**Figure 1:**
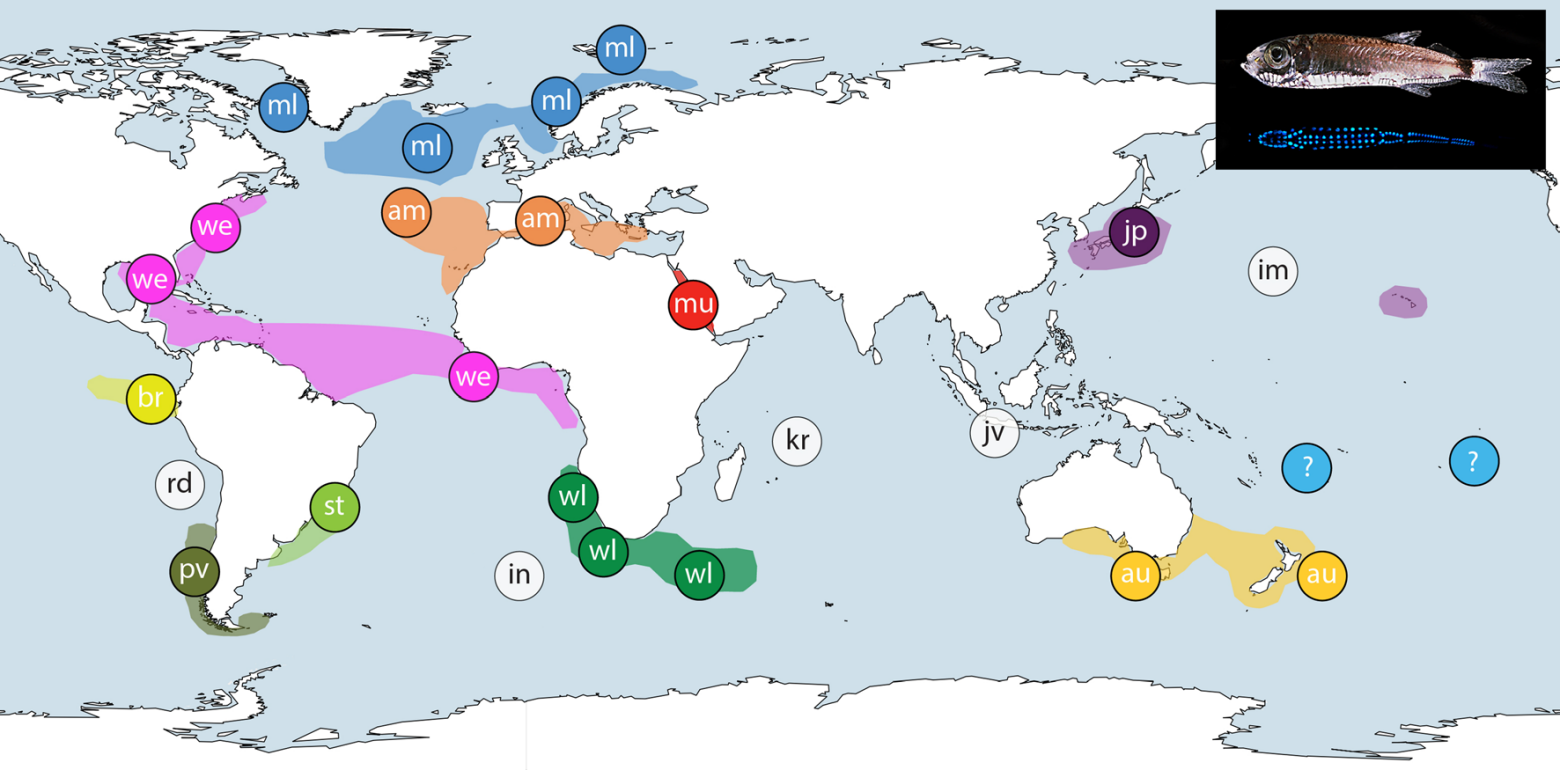
Map of *Maurolicus* distribution and sampling locations. Sampling locations with described species are indicated by a two-letter code. Distributions represented display literature and museum catalogue data as presented by Parin and Kobyliansky (1996); species described by those authors are indicated with an asterisk below. ml: *M. muelleri* (Gmelin, 1789), am: *M. amethystinopunctatus* Cocco, 1838, mu: *M. mucronatus* Klunzinger, 1871, we: M. weitzmani*, st: M. stehmmanni*, wl: M. walvisensis*, au: *M. australis* Hector, 1875, jp: *M. japonicus* Ishikawa, 1915, pv: *M. parvipinnis* Vaillant, 1888, br: M. breviculus*. The unknown *Maurolicus* species from Fiji, French Polynesia and American Samoa is indicated with a question mark. Unsampled species and their type localities are indicated with white circles; rd: M. rudjakovi*, in: M. inventionis*, kr: M. kornilovorum*, jv: M. javanicus*, im: M. imperatorius*. Inset A shows *Maurolicus muelleri* together with a ventral view of the bioluminescent light organs under UV-light. The base map was constructed from the GSHHG World Vector Shoreline data set (WVS; Version 2.3.7) using the open source QGIS^19^. Symbols and the image of *Maurolicus* were added using Adobe Illustrator.

Samples of *Maurolicus* were obtained from a number of locations and institutions for inclusion in this study with details of material presented in Table 1. Further sample details, including latitude and longitude, are included as part of relevant GenBank accessions. A graphical overview of specimen locations together with published ranges of described species (following Parin and Kobyliansky^12^) is shown in Fig. 1. No live fish were involved in this work, which utilised previously collected ethanol-preserved specimens. Specimens were fixed in 96% ethanol, either directly after collection or subsequent to storage at − 20 °C. The majority of material comprised adult *Maurolicus* specimens but for some locations we made use of whatever samples were available, such as larvae (Chile) and fragments of recently ingested *Maurolicus* taken from bigeye tuna stomachs (Fiji and French Polynesia).

**Table 1:** Sampling locations for putative *Maurolicus* species, with an overview of GenBank and BOLD accession numbers for COI and ITS-2 sequences used in analyses.

Putative species	Sampling locations	COI accessions	ITS-2 accessions
*M. muelleri*	Central North Atlantic (24/21), Norway (25/25), Greenland (1/0)	new MT132216, MT132223-132,224, MT132234, MT132238-MT132251, MT132253-MT132261, MT132263-MT132264, MT132271-MT132273, MT132304-MT132313 also KU958034, KU958039, KU958038, KU958033, EU148245-EU148247, BOLD: GLF028-13	new MT132801, MT132806, MT132809, MT132814-MT132818, MT132820-MT132827, MT132829-MT132839, MT132842-MT132844, MT132873-MT132881 also KU958083, KU958084, KU958089, KU958092, KU958094, KU958087, KU958091, KU958086
*M. amethystinopunctatus*	Azores (8/8), Mediterranean Sea (18/10)	new MT132252, MT132262, MT132270, MT132274-MT132284, MT132298 also KU958035-KU958037, KC616398-KC616403, KJ709557-KJ709558,	new MT132800, MT132819, MT132828, MT132845-MT132855, MT132859, MT132867 also KU958085, KU958088
*M. mucronatus*	Red Sea (8/6)	new MT132322-MT132329	new MT132888-MT132893
*M. weitzmani*	Liberia (8/8), Gulf of Mexico (10/6), Western North Atlantic (7/6) [plus two Genbank sequences with no location given]	new MT132197-MT132203, MT132225-MT132228, MT132265-MT132269 also KU958021–KU958025, GQ860362, KF930110, KJ190037, MF041314, MF041576, KX098554	new MT132783-MT132788, MT132807-MT132808, MT132810-MT132811, MT132840-MT132841 also KU958095–KU958102
*M. walvisensis*	Namibia (10/10), South Africa (12/11), Southwest Indian Ridge (5/4)	new MT132285-MT132297, MT132299-MT132303, MT132317-MT132321 also KU958026, KU958028,KU958029, KU958031	new MT132856-MT132858, MT132860-MT132866, MT132868-MT132872 also KU958073, KU958080, KU958082, KU958075, KU958076, KU958090
*M. australis*	New Zealand (10/10), Tasmania (10/6) [plus one Genbank sequence with no location given]	new MT132217-MT132222, MT132229-MT132233, MT132235-MT132237, MT132314-MT132316 also KU958030,KU958032, KU958027, GQ860361	new MT132802-MT132805, MT132812-MT132813, MT132882-MT132883 also KU958078, KU958081, KU958093, KU958072, KU958074, KU958077, KU958079
*M. japonicus*	Japan (12/11) [plus one Genbank sequence with no location given]	new MT132204-MT132215 also KU199192-KU199196	new MT132789-MT132799
*M. stehmanni*	Brazil (8/8)	new MT132177-MT132184	new MT132767-MT132774
*M. parvipinnis*	Chile (5/3)	new MT132185-MT132189	new MT132775-MT132777
*M. breviculus*	Galápagos Islands (2/3)	new MT132196 also BOLD: LIDMA1145-12	new MT132780-MT132782
*M.* sp.	Fiji (4/2), French Polynesia (2/0), American Samoa (1/0)	new MT132176, MT132190-MT132195	new MT132778-MT132779

Numbers of sequences per sampling location are given in parentheses for COI and ITS-2, respectively. Further details for new material, including latitude and longitude, are included as part of GenBank accessions.

### DNA extraction, amplification and sequencing

Genomic DNA was extracted from ca. 2 mm^3^ of ethanol preserved muscle tissue (or whole individuals in the case of small larvae) using the QIAamp DNA Mini Kit (Qiagen, Oslo, Norway) following the manufacturer’s standard protocols. Mitochondrial COI and nuclear ITS-2 genes were amplified by polymerase chain reaction using previously published primers and PCR conditions as contained in Rees et al.^13^. Internal COI primers were designed for *Maurolicus* samples recovered from tuna stomachs after these failed to amplify using standard amplification procedures (MA_SPAC_F1: GCGGCTTTGGAAACTGATTA; MA_SPAC_R1: TCCTGCAAGAGGAGGGTAGA; MA_SPAC_F2: GGGGACGACCAAATCTACAA and MA_SPAC_R2: GCGAGCAGAAGAAGGAAAGA.). A short fragment of COI was subsequently sequenced for four specimens from Fiji (177 bp) and two overlapping fragments sequenced for two samples from French Polynesia (yielding 258 bp). PCR products were visualised on 1% agarose gels and stored at 4 °C until purification and sequencing. Sequence reactions were performed using the BigDye v3.1 Cycle Sequencing Kit (Applied Biosystems, Inc., Norwalk, CT, USA) with the same primers used for PCR amplification. Both strands of all PCR products were sequenced using an ABI 3730 capillary sequencer at UiB.

### Phylogenetic analyses

A total of 53 new COI and 43 new ITS-2 sequences were generated for this study. Additional sequences were available from a previous study on *Maurolicus* by one of the authors^13^ and these were supplemented by COI sequences from GenBank (n = 23) and BOLD (n = 2) databases, giving a total of 198 and 157 ingroup sequences for COI and ITS-2, respectively. Full sequence details and accession numbers are presented in Table 1. For COI, *Vinciguerria poweriae* (MT128722) was used as an outgroup, along with additional sternoptychid sequences obtained from GenBank. For ITS-2 we used *Anoplopoma fimbria* (AB244631) as in our previous *Maurolicus* study (Rees et al. 2017). DNA sequences were aligned with MUSCLE^20^ implemented in MEGA v7.0.26^21^. Best-fit models of nucleotide substitution were inferred for both COI and ITS-2 using JModeltest v2.1.4^22,23^. Both Akaike information criterion (AIC) and Bayesian information criterion (BIC) results indicated the best-fit model to be GTR + I + G for COI and GTR + G for ITS-2. Maximum likelihood (ML) and Bayesian Inference (BI) methods were used both for unique haplotype and full datasets in order to identify well-supported clades representing potential species under the phylogenetic species concept (i.e. molecular operational taxonomic units; MOTUs^24^). For the ITS-2 data, ML and BI analyses were run with gap-forcing sites included and removed.

ML analyses were performed with PhyML^22,25^ implemented in Seaview^26^ and the GTR + I + G model with six rate categories and tree search operations using the Best of NNI & SPR option. Nonparametric bootstrapping was used to assess clade support (100 replicates) and only values above 80% were considered significant^27^. MrBayes v3.2^28^ was used to execute BI analyses of both single-gene and concatenated mitochondrial + nuclear datasets, the latter comprising all observed unique COI and ITS-2 haplotypes. Markov-chain Monte Carlo analyses consisted of two independent runs, each consisting of four chains and running for 10 million generations, sampling every 1,000 generations. Results were visualised in Tracer v. 1.5.0^29^ and proper mixing of the MCMC was assessed by calculating the effective sampling size (ESS) for each parameter. For each data set, the maximum clade credibility tree (MCC; the tree with the largest product of posterior clade probabilities) was selected from the posterior tree distribution (after removal of 25% burn-in) using the program TreeAnnotator version 1.8.0 (available as part of the BEAST package; v2.1.1^30^). Bayesian posterior probability (BPP) values of 95% or higher were considered significant^31^.

### Species delimitation

Assessment of possible species validity was explored through four methods, using the COI dataset for comparisons with MOTUs identified through phylogenetic analyses. Two methods used pairwise sequence distances between specimens to determine the number of OTUs: (1) Automatic barcode gap discovery (ABGD^32^) and (2) statistical parsimony analysis (TCS^33,34^) and the other two are based on coalescent theory: (3) Generalised mixed Yule coalescent (GMYC^35^) and (4) Bayesian Poisson tree processes (bPTP^36^). These contrasting approaches were used in an attempt to gain a robust estimate of potential species groupings in our *Maurolicus* dataset through congruence of delimitation across methods.

ABGD delimits sequence data into putative species based on the so-called “barcode gap” between intraspecific and interspecific pairwise differences. A range of prior intraspecific divergence is used to infer a limit for intraspecific divergence from the dataset and the barcode gap, detected as the first significant gap beyond this limit, is then used to partition the data. Following initial partitioning a second round of splitting (recursive partitioning) is performed. ABDG analysis was carried out using the web version (http://wwwabi.snv.jussieu.fr/public/abgd/abgdweb.html) using the K2P model to calculate pairwise distances (K80 option with TS/TV = 2.0), 20 recursive steps and other parameters at default settings (Pmin 0.001, Pmax 0.1, Nb bins (for distance distribution) = 20, Relative Gap width 1.5). Statistical parsimony networks have proved to be a useful approach for assessing biological species diversity, with sub-networks generated from COI data found to be largely congruent with Linnean species^37^. We used the TCS software (v.1.21)^34^ to perform this analysis, which calculates the probability of parsimony^33^ for pairwise sequence differences until a default 95% cutoff value is reached. The number of mutational differences associated with the cutoff probability is then used as the maximum number of mutational connections between pairs of sequences, allowing generation of haplotype networks justified under the parsimony criterion. Analysis was run on the full COI dataset, with gaps treated as missing data.

GMYC is a model-based approach that determines a threshold value marking the transition from speciation processes to coalescent population processes on an ultrametric tree, using time to identify branching rate transition points. In order to test effects of different input trees on GMYC we generated four Bayesian inference trees using (1) a Yule model with a constant clock, (2) a Yule model with a relaxed clock, (3) a coalescent model with a constant population size and a constant clock and (4) a coalescent model with a relaxed clock. Ultrametric trees comprising all unique *Maurolicus* COI sequences were generated using BEAST, with priors set in BEAUti, and analysed with GMYC as implemented in the SPLITS package (v. 2.10)^38^ in R^39^. The second coalescent theory method used, bPTP, requires an estimated gene tree with branch lengths proportional to the amount of genetic change. Branch lengths are used to estimate the average number of substitutions per site between two branching events, and a significantly higher number of substitutions are assumed to be present between species than within species^36^. bPTP adds Bayesian support values to delimited species on the input tree and higher values on a node indicate that all descendants from this node are more likely to be from one species. For bPTP analyses we used the web-based server (https://species.h-its.org/)^36^, utilising an initial Newick-format PhyML topology rooted on *Vinciguerria poweriae*. Other settings were retained as default (100,000 MCMC generations, Thinning 100, Burn-in 0.1).

## Results

### Sequence data

The final aligned COI dataset consisted of 684 bp for 198 ingroup taxa, with 51 unique haplotypes. After alignment of the 157 ITS-2 sequences, that dataset comprised 410 bp; removal of 138 gap-forcing sites resulted in a dataset of 272 bp, with 38 and 20 unique haplotypes, respectively. Material collected and sequenced for this study expanded our geographic sampling to new locations including the Northwest Pacific (off Japan; 12 specimens sequenced for COI, 11 for ITS-2), the Red Sea (8 COI, 6 ITS-2), off Brazil (8 COI, 8 ITS-2), over the Southwest Indian Ridge (SWIR; 5 COI, 4 ITS-2), Gulf of Mexico (7 COI, 6 ITS-2), off Chile (5 COI, 3 ITS-2), off the Galápagos Islands (1 COI, 3 ITS-2) and the South Pacific off Fiji, French Polynesia and American Samoa (7 COI, 2 ITS-2). Some *Maurolicus* samples taken from bigeye tuna stomachs proved problematic for DNA amplification; two of four samples from off French Polynesia were successfully sequenced for a short fragment of COI but failed for ITS-2; two of the four samples from off Fiji were sequenced for ITS-2 along with all four samples for COI. All COI sequences for these specimens were identical to a previous sequence for a *Maurolicus* specimen collected from north of American Samoa by Jan Yde Poulsen (JYP_001/1380_MASP: MT132176).

### Phylogenetic analyses

**Figure 2:**
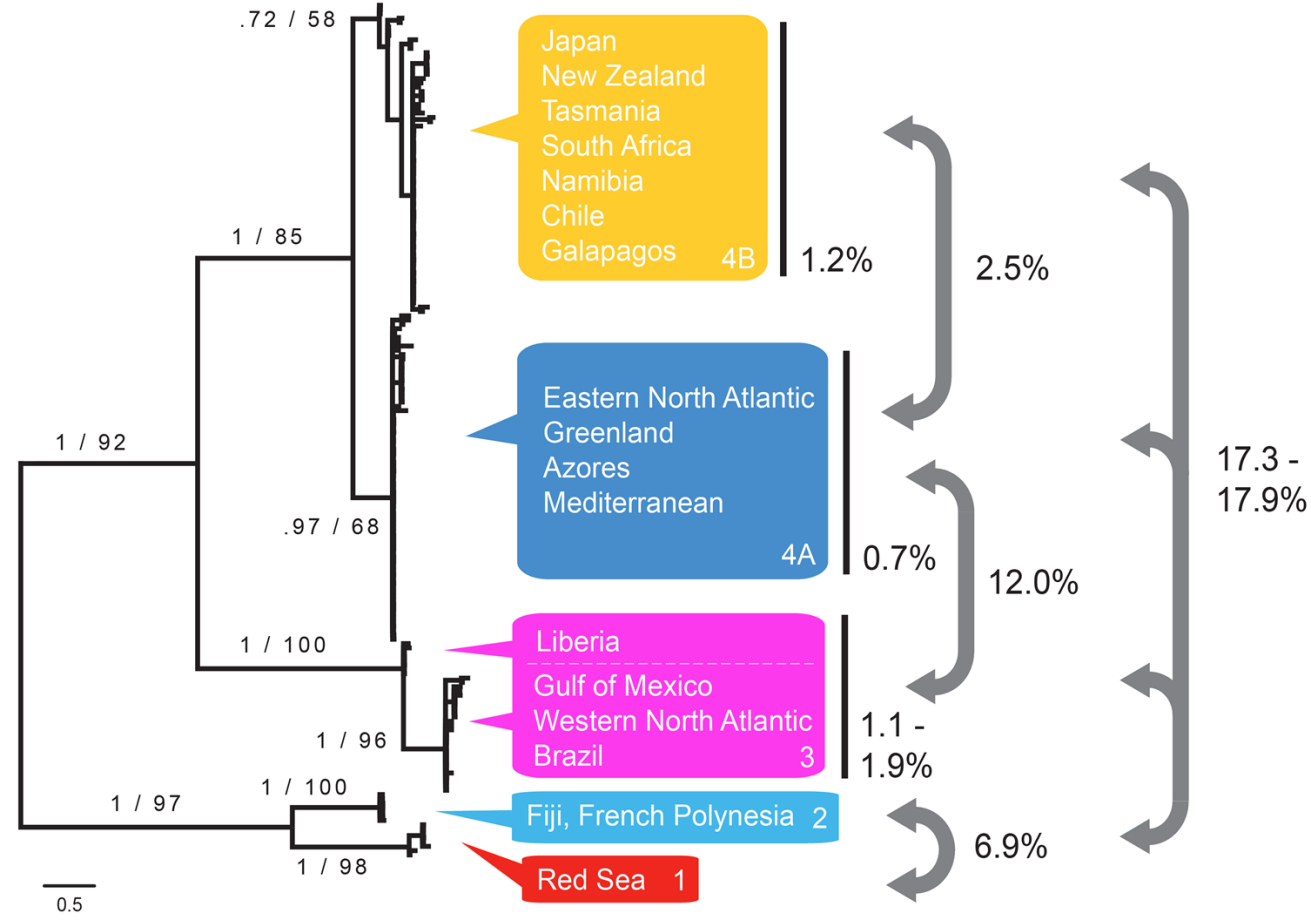
Bayesian phylogeny based on COI data (full 198 sample dataset). Bayesian posterior probabilities and ML bootstrap support values (from ML analysis of 157 *Maurolicus* sequences) are indicated for the various clades. Geographical locations of specimens within clades are shown together with maximum p-distances next to white bars (minimum and maximum p-distances are shown for eastern Equatorial vs. western Atlantic / Gulf of Mexico). Relationships among groups are shown by mean between-group p-distances.

**Figure 3:**
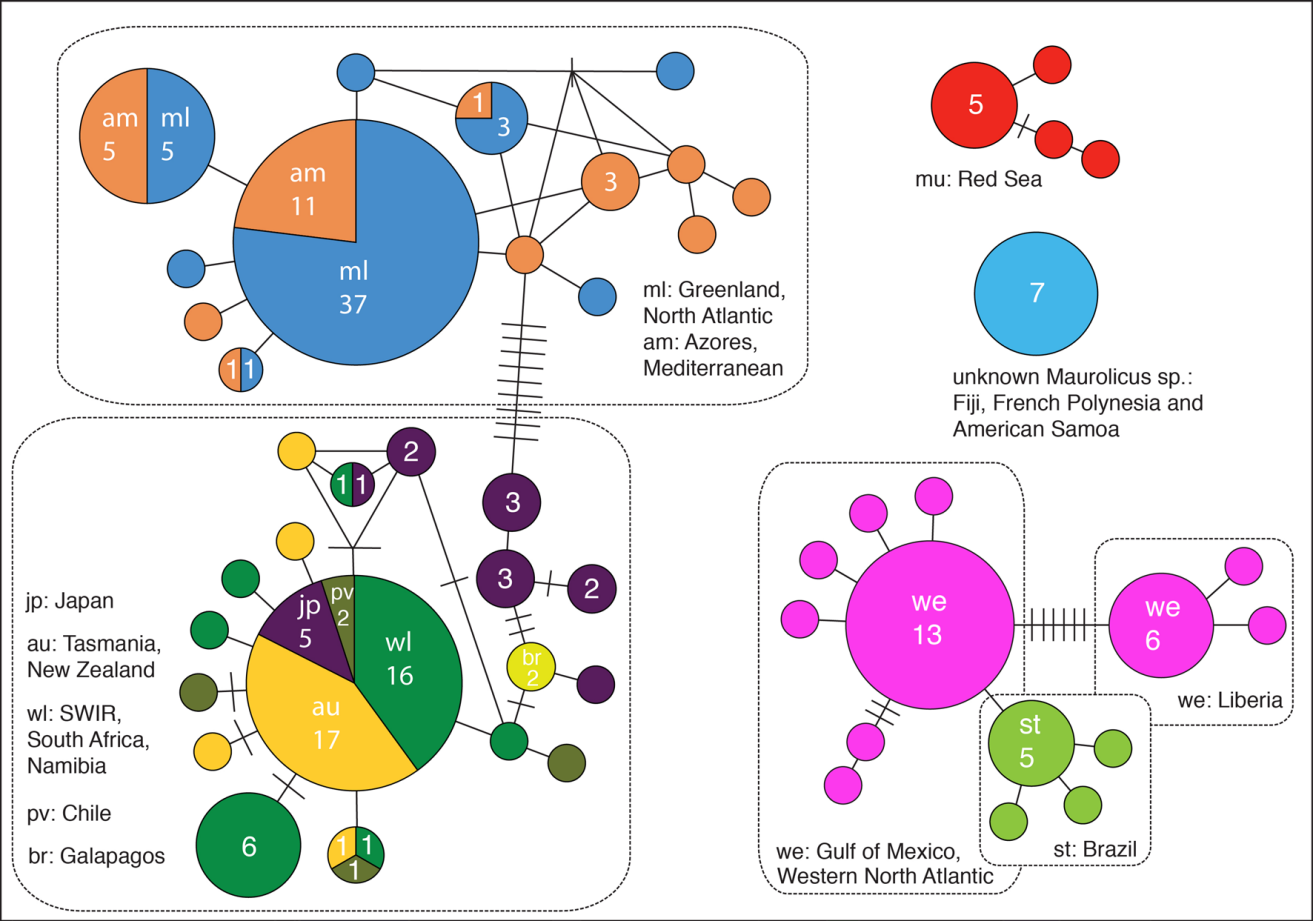
Statistical parsimony haplotype network resulting from analysis of *Maurolicus* COI data. Putative species are colour coded with the same coding applied to the sampling and distribution map (Fig. 1). Circle size indicates haplotype frequency; frequency of haplotypes is also presented, with a breakdown for those shared between multiple sampling locations. Small circles with no number given represent unique haplotypes. Branches connecting haplotypes within a subnetwork represent single nucleotide substitution steps; bars crossing these branches represent additional substitution steps (i.e. missing haplotypes).

All phylogenetic analyses recovered the same basic topology with very clear geographical groupings (Figs. 2 and 3). Four geographically distinct lineages with consistently high support were recovered in all COI and combined COI + ITS-2 analyses, whether based on the full dataset or only unique haplotypes: (Clade 1) the Red Sea; (Clade 2) waters off Fiji and French Polynesia; (Clade 3) western North Atlantic, Gulf of Mexico, Brazil and the eastern equatorial Atlantic; and, (Clade 4) the Mediterranean Sea, the central and eastern North Atlantic, the western North Pacific, and the South Atlantic, Pacific, and Indian Oceans. Clade 4 is further split into two geographically distinct clades with moderate to high support in Bayesian and Maximum Likelihood analyses: (4A) a clade containing all *Maurolicus* from the Mediterranean Sea, the central and eastern and far northern North Atlantic; and (4B) a mainly southern-hemisphere group, comprising *Maurolicus* from Japan and all southern Atlantic and Pacific sampling locations with the exception of specimens from off Brazil, Fiji, French Polynesia and American Samoa.

An overview of within- and between-clade genetic distances for the COI data is presented in Fig. 2. Pairwise genetic distances reveal different levels of divergence within the genus *Maurolicus*, with low differentiation exhibited by clades covering an enormous geographical range contrasted by shallow to deep divergences between lineages. Specimens in Clade 3 exhibited low levels of genetic variation, with the highest differentiation between the eastern equatorial Atlantic (off Liberia) and the western Atlantic material (1.9%). For the western Atlantic samples, spanning a range from southern Brazil to the Mid-Atlantic Bight, the maximum p-distance was 1.1%. Similarly, the maximum uncorrected p-distance for specimens encompassing a range from Japan to New Zealand, South Africa, Chile and the Galápagos was 1.2% (clade 4B). Furthermore, the TCS network showed an identical COI haplotype that was shared by many individuals from across this distribution. A relatively shallow divergence (2.5%) was found between this predominantly southern hemisphere clade and the central/eastern North Atlantic clade (4A). Maximum differentiation within the latter clade was low (0.7%). Deeper divergences appeared to exist between the other clades, ranging from 6.9% between the South Pacific and Red Sea (Clades 1–2) to 12.0% (western/equatorial Atlantic vs. central/eastern North Atlantic; Clades 3–4) to over 17% between the basal clades (1 and 2) and the other *Maurolicus* lineages (Clades 3, 4A and 4B).

**Figure 4:**
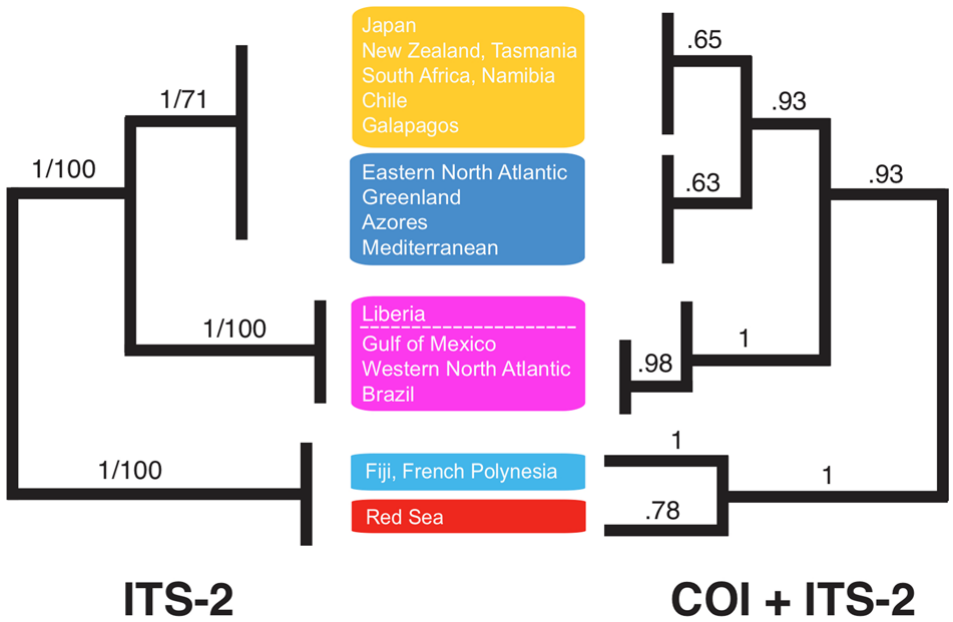
Topologies resulting from maximum-likelihood and Bayesian analyses of *Maurolicus* for ITS-2 and concatenated COI + ITS-2 data. Bootstrap support (ML) and Bayesian posterior probability values are indicated.

Analyses using ITS-2 data alone were congruent with patterns seen with COI and combined analyses, albeit with the lower variability of this marker resulting in reduced resolution for more recently diverged clades. All ITS-2 analyses (all sequences vs. unique haplotypes, full dataset vs. gap-forcing sites removed) yielded identical topologies in both Maximum Likelihood and Bayesian reconstructions, with three distinct lineages: (1) South Pacific and Red Sea specimens, (2) western and equatorial Atlantic, and (3) all other *Maurolicus* from the North Atlantic and widespread, predominantly southern hemisphere COI clades (Fig. 4). Maximum uncorrected p-distances (not counting gap vs. non-gap sites as differences) within the three clades were 0% for the Red Sea and Fiji (two haplotypes from eight individuals, differing only by a 3 bp indel), 3.6% for the western and equatorial Atlantic (11 haplotypes from 28 individuals, with no geographic structure evident), and 1.6% for the remaining material from the central/eastern North Atlantic and “southern hemisphere + Japan” clade (25 haplotypes from 121 individuals). Minimum and maximum between-clade distances were as follows: North Atlantic/southern hemisphere vs. western and equatorial Atlantic: 10.9–13.4%, North Atlantic/southern hemisphere vs. South Pacific/Red Sea: 15.2—16.8%, and western and equatorial Atlantic vs. South Pacific/Red Sea: 16.5–18.9%.

### Species delimitation

**Figure 5:**
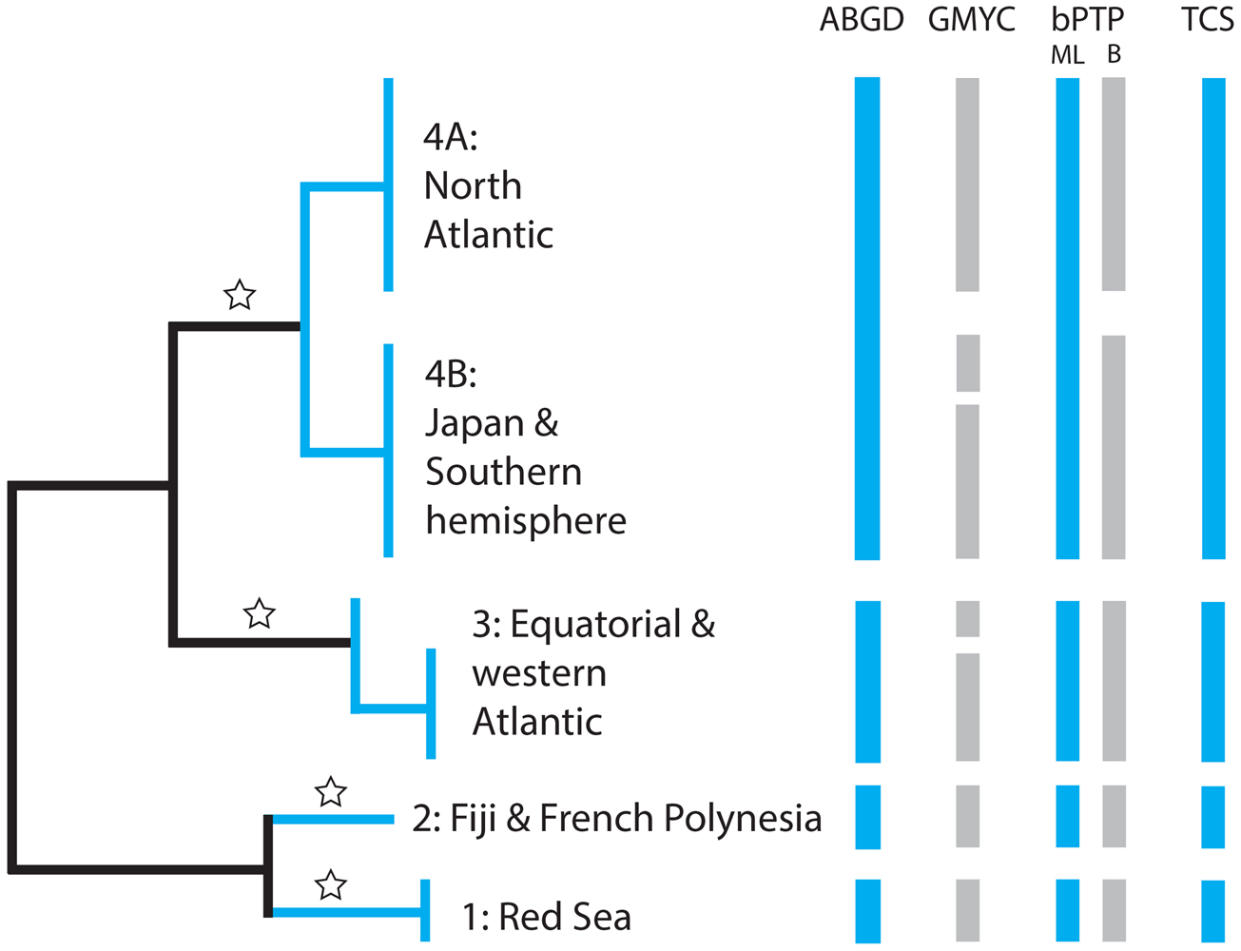
Overview of groupings resulting from the various species delimitation methods applied to COI data. A representative, simplified topology (combined COI + ITS-2) is shown; the four strongly supported clades are indicated with a star and basic geographical ranges of clades are indicated. Vertical bars indicate putative species groups for each method. Groupings from GMYC are shown for Yule and coalescent models with a constant clock. Results for bPTP from Maximum Likelihood (ML) and Bayesian (B) partitions are indicated.

Our species delimitation analyses aim to identify putative species, ideally identifying lineages consistent across multiple methods. These putative species require further validation through additional lines of evidence such as multi-gene sequence data and morphological characters. An overview of the results of the various species delimitation approaches is presented in Fig. 5. Three of the four methods (ABGD, Maximum Likelihood solution bPTP and TCS) applied to our COI dataset resulted in the same putative species groupings, in agreement with the four well-supported clades in our phylogenetic analyses (COI and combined dataset Clades 1–4). ABGD analysis resulted in 12 sequence partitions with identical output for both initial and recursive partitions. The first two partitions (*P* = 0.0010 and 0.0013) resulted in oversplitting, each with 44 MOTUs, while partition 12 contained just one MOTU (*P* = 0.0144). Otherwise, results from ABGD were very stable, with partitions 3 to 11 all producing four MOTUs (*P* = 0.0016 to 0.0113) corresponding to the highly supported Clades 1 to 4 in our phylogeny.

The statistical parsimony network analysis performed with TCS resulted in four unconnected networks with each haplogroup again consistent with the four major geographically distinct lineages described previously (Fig. 3). Of the 51 COI haplotypes identified at the 95% connection limit, 11 were only present in sequences from GenBank and one from BOLD (Greenland specimen). The first two haplogroups have narrow geographical ranges (corresponding to Clades 1 and 2; the Red Sea and the South Pacific) while the other two are far more widespread. The third haplogroup contains *Maurolicus* from the eastern equatorial and western Atlantic, while the fourth contains individuals collected from all World Ocean divisions except for the Southern Ocean. The most common haplotype for Clade 3 was shared by individuals from the Gulf of Mexico and the Mid-Atlantic Bight, with specimens from off southern Brazil differing from this by 1–2 substitutions. Eastern equatorial Atlantic specimens were also distinct, differing from the majority haplotype by 7–8 substitutions. The shallow divergence between subclades 4A (central/eastern Atlantic and Mediterranean) and 4B (southern hemisphere + Japan) is again reflected by the TCS analysis. No shared COI haplotypes were observed between these subclades. The closest haplotypes from these geographically consistent subclades differed by 10 substitutions. Although all sequences from the combined Clade 4 formed a single subnetwork, the observed pattern was of a majority haplotype for both subclades 4A and 4B, with a number of less common or unique haplotypes differing from the majority by 1–3 steps for Clade 4A (14 haplotypes) and for 1–8 steps for Clade 4B (18 haplotypes). The majority COI haplotype in Clade 4A is found in the Mediterranean Sea and the North Atlantic from the Azores to Arctic Norway, while that in 4B is shared by individuals collected off Japan, New Zealand, Tasmania, the Southwest Indian Ridge, South Africa, Namibia and Chile.

For the GMYC analysis, the Maximum Likelihood of the null model (uniform branching across the tree, indicating all sequences form a single cluster) was significantly lower than the GMYC model for three of the four prior tree settings tested (Table 2), with putative species entities broadly conforming to those emerging from other methods. Both Yule and coalescent models with a constant clock gave the same six clusters and seven ML entities (i.e. including singletons). The differences between these seven putative species and the four clades in the phylogenetic analyses are the splitting of: (1) eastern equatorial Atlantic *Maurolicus* from the western Atlantic, (2) the North Atlantic and the predominantly southern hemisphere material, and (3) three of the Japanese haplotypes from the remainder, which remain grouped with the rest of the material from multiple regions within that clade. For the coalescent model with a relaxed clock, eight clusters and nine entities were identified, with the same splitting as for the seven-entity results but with additional division of North Atlantic material into two entities (with no geographical pattern) and splitting of the predominantly southern hemisphere material into three entities (Japan, Japan + Galápagos, and all other material, including more haplotypes from Japan). Employing a Yule model with a relaxed clock gave 11 clusters and 14 entities but with a non-significant result for the likelihood ratio test.

**Table 2:** Results of generalised mixed yule coalescent (GMYC) analyses of COI data.

Tree prior	Clusters	Species	CI	L-null	L-GMYC	LRT
Yule, constant clock	6	7	6–13	357.660	371.066	< 0.000
Yule, relaxed clock	11	14	2–20	349.493	351.555	0.127 n.s
Coalescent, constant	6	7	5–15	370.612	382.075	< 0.00
Coalescent, relaxed clock	8	9	6–16	370.465	381.486	< 0.000

Clusters, OTUs identifed by GMYC containing more than one specimen; Species, number of ML entities (includes singletons); CI, confidence interval for number of entities; L-Null, likelihood of the null model (i.e. all sequences form a single cluster); L-GMYC, likelihood of GMYC model; LRT, *P*-value of likelihood ratio test.

In bPTP analyses, four and five putative species were recovered by Maximum Likelihood and Bayesian partitions, respectively (excluding outgroup). In both cases, Red Sea and the South Pacific were highly supported (support = 1.000). The remaining putative species had lower support, indicating difficulty in delimiting species in these cases. Clade 3 (eastern equatorial and western Atlantic) was consistent in both partitions (ML and Bayesian support = 0.660) while Clade 4 (ML support = 0.496) was split along the lines of subclades 4A and 4B (i.e. central/eastern North Atlantic + Mediterranean vs. predominantly southern hemisphere + Japan) in the Bayesian partition (support = 0.504 for both putative species).

## Discussion

The findings of this study clearly suggest that multiple described species of *Maurolicus* group together to form cosmopolitan mesopelagic taxa, rather than discrete allopatric entities, as has previously been reported. Our phylogenetic analyses of *Maurolicus* on a global scale indicate four main groups with clear geographical subdivision (Figs. 2 and 3). Within Clade 4 there is a relatively shallow divergence between a central/eastern North Atlantic clade (4A) and a cosmopolitan ‘southern’ clade (4B: South Pacific, South Atlantic, and Indian Oceans + Northwest Pacific). Although species delimitation analyses suggest that this group may be a single species, there is some degree of separation between the North Atlantic / Mediterranean and the predominantly southern hemisphere group and we propose maintenance of *M. muelleri* for the North Atlantic / Mediterranean (previously two species) and *M. australis* for the southern hemisphere (previously five species). These clades correspond to the ‘northern’ and ‘southern’ clades identified by Rees et al.^13^ but with a significant geographic expansion of the southern hemisphere clade and an extension of the northern group to include *Maurolicus* from western Greenland. Our data indicate that the geographical range for the ‘southern’ clade encompasses *Maurolicus* from Chile and the Galápagos Islands. Within this group the majority COI haplotype was shared by specimens from across the entire range (with the exception of the Galápagos, from which only a small number of samples were available) and low genetic divergence was observed for the clade as a whole (maximum COI p-distance 1.2%).

For the ‘southern’ clade (Clade 4B), a lack of genetic and morphological differentiation between North Pacific *M. japonicus*, South Pacific / Indian Ocean *M. australis* and South Atlantic *M. walvisensis* has been noted previously, with studies proposing synonymisation of *M. walvisensis* and *M. japonicus*^16,17^ and a subsequent expansion to include both of these as junior synonyms of *M. australis*^13,18^. On a genetic basis, this group is now expanded to include two eastern Pacific species, *M. breviculus* and *M. parvipinnis*. Furthermore, several other species have been described with very restricted distributions that lie within the geographical range of the ‘southern’ clade. In the North Pacific, *M. imperatorius* is associated with the Emperor Seamount, with populations of *M. japonicus* found to the west around Japan and east around Hawaii. In the light of our data, it seems unlikely that distinct *Maurolicus* species persist on these seamounts and it is instead perhaps more probable that these represent an additional population of the widespread ‘southern’ clade *Maurolicus*.

**Figure 6:**
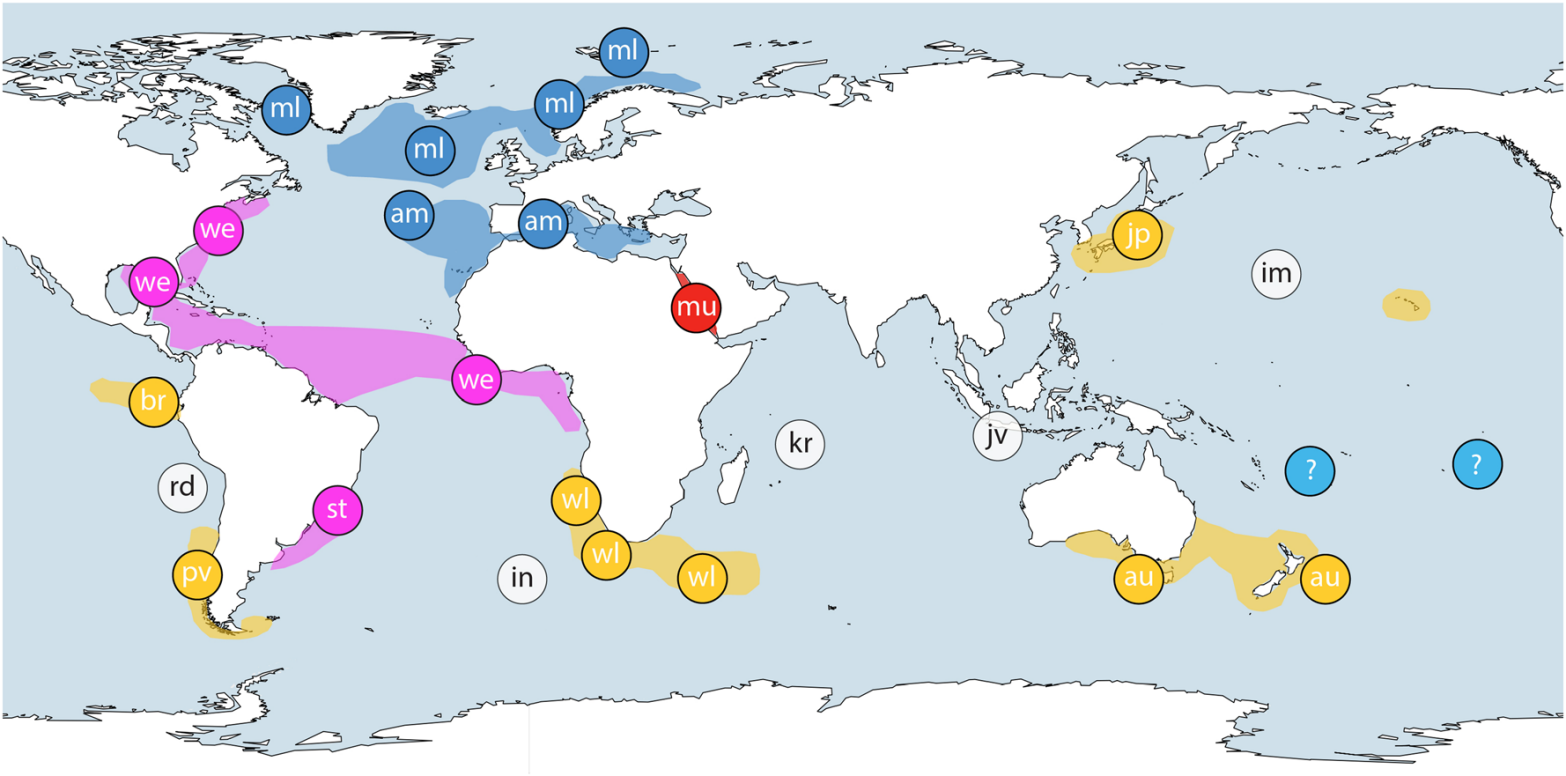
Revised map of *Maurolicus* species distribution. Representation of current genetic groupings for *Maurolicus*, colour coded to reflect COI haplotype groupings and suggested putative species: (1) *M. muelleri*; (2) *M. australis*; (3) *M. mucronatus*; (4) *M. weitzmani* and (5) a presently uncharacterised *Maurolicus* species from the central South Pacific. Unsampled species are indicated as in Fig. 1. The base map was constructed from the GSHHG World Vector Shoreline data set (WVS; Version 2.3.7) using the open source QGIS^19^. Symbols were added using Adobe Illustrator.

A similar situation may apply to *M. inventionis* in the South Atlantic and *M. rudjakovi* in the eastern South Pacific. Both species are described with distributions restricted to seamounts (Discovery Seamount and seamounts of the Nazca Ridge, respectively) adjacent to or nested within described ranges of other taxa now identified as part of the widespread southern hemisphere clade. Populations of *Maurolicus* on seamounts in the South Atlantic have been reported to comprise mainly immature specimens^40^ and it has been suggested that these rely on advection from populations near Argentina and Tristan da Cunha Island^41^. Whether such populations are self-sustaining or require recruitment from neighbouring populations is unknown. Taking this further, of the five described species we have been unable to include in our work to date (white circles in Figs. 1 and 6), four have very localised distributions (seamounts or, in the case of *M. kornilovorum*, Saya de Malha Bank in the Indian Ocean).

The shallow divergence between the ‘southern’ and North Atlantic / Mediterranean clades was reflected by bPTP (Bayesian) analysis, with low support for these as two putative species. GMYC analysis also resulted in putative species for the ‘northern’ and ‘southern’ clades but also split three Japanese haplotypes from the southern clade into a separate species group, which would seem to emphasise the tendency of this method towards oversplitting (e.g.^32,35^.) and limit confidence in this result. The ‘northern’ and ‘southern’ *Maurolicus* clades, previously found by Rees et al.^13^ to form unconnected groups in TCS haplotype networks based on COI data, now form a single subnetwork due to ‘bridging’ haplotypes stemming from inclusion of material from Japan (Fig. 4). However, despite extensive sampling, no shared haplotypes were found between *Maurolicus* from these two regions and the most similar haplotypes from the two remain separated by 10 substitutions (Fig. 4). Since no shared haplotypes were found between the North Atlantic and ‘southern’ clades, it may be that our results reflect the difficulty of species delimitation methods in dealing with species with a shallow divergence.

*Maurolicus weitzmani* from the equatorial and northwest Atlantic, together with *M. stehmanni* from the western South Atlantic, also form a single putative species (Clade 3), which we propose are synonymised as *M. weitzmani*. A single species was recovered in almost all species delimitation analyses (Fig. 5). The exception was in the GMYC analysis, where the eastern equatorial samples were differentiated from the western Atlantic *Maurolicus*. A significant geographical distance was initially reported between the distributions of *M. stehmanni* (the continental slope of the western South Atlantic, between 34° and 40° S) and *M. weitzmani* (western North Atlantic to the Gulf of Mexico and Equatorial Atlantic from 45° W to slopes of Africa^12^). However, subsequent studies of *M. stehmanni* extended the range of this putative species to 11° S in the western Atlantic^14^, significantly narrowing the gap between the known distributions of the two species described for the western Atlantic. COI sequences for *Maurolicus* from off Brazil are relatively indistinct from other populations in this clade, differing by only one to two substitutions (compared with a minimum of seven substitutions [1.9%] between the western and eastern equatorial Atlantic; Fig. 4).

The remaining two clades (1 and 2) represent a highly divergent lineage containing two distinct taxa, one from the Red Sea (*M. mucronatus*) and one from the central South Pacific (sp. undetermined). *Maurolicus mucronatus* Klunzinger, 1871 (Clade 1) represents one of the earlier described species in the genus; this species is restricted to the Red Sea and is clearly distinct in all species delimitation estimates based on COI data (Fig. 5). Equally distinct is the *Maurolicus* species from the central South Pacific (Clade 2), which is deeply divergent from all other main Clades and is clearly identified as a putative species in all analyses. Our material appears to represent the first records in the region for this genus. Known distributions based on museum collections, published data and specimens examined by Parin and Kobyliansky^12^ (see overview in Fig. 1) indicate no known *Maurolicus* species present in this area. Without material to perform adequate morphological analyses we are unable to formally characterise the central South Pacific *Maurolicus*; presently, we have just one complete specimen available. However, molecular and distribution data suggest that this may represent an undescribed species.

The geographic range of the central South Pacific *Maurolicus* is unknown but our sampling includes Fiji, French Polynesia and American Samoa. Since the *Maurolicus* from Fiji and French Polynesia were obtained from bigeye tuna caught with longlines it is worth commenting on the certainty of the probable locations of where these *Maurolicus* were ingested relative to where the tuna were caught. Prey as small as *Maurolicus* are likely to be fully digested by tuna within 4–5 h; our samples were relatively intact and, based on average swimming speeds and allowing for the unknown position of the exact point where the tuna were caught on the longline, ingestion was estimated at 100–150 km from the catch location (Valerie Allain, pers. comm.). A number of described *Maurolicus* species are found in areas adjacent to, but at significant distance from, this region; to the north (*M. japonicus* and *M. imperatorius*), south (*M. australis*), west (*M. javanicus*) and east (*M. breviculus*, *M. rudjakovi*, *M. parvipinnis;* see Fig. 1). Four of these seven putative species have been included as part of this study and form part of the genetically indistinguishable ‘southern’ clade while two others have very restricted ranges that lie nested within sampled species’ distributions and that we therefore consider to have somewhat doubtful status (Figs. 1 and 6; see discussion below). The last remaining species is *M. javanicus*, reportedly distributed in the eastern tropical Indian Ocean off Java and Australia and the northern Coral Sea^12^. At present, we cannot rule out that our central South Pacific *Maurolicus* may represent an extended distribution for a described species that has not been sampled—*M. javanicus* being the most likely candidate. To examine this possibility, we would need samples collected from within the described range of *M. javanicus* with which to compare with our central South Pacific material.

Our data indicate a lack of support for maintenance of many of the currently described allopatric species in this genus. Conversely, members of *Maurolicus* appear to be true eucosmopolitan species^42^—taxa with a natural and prehistorically global (or extremely broad) distribution. Figure 6 presents a summary of geographic groupings based on our species delimitation analyses and subsequent recommendations, updating the distribution information presented in Fig. 1. More data are required to evaluate levels of gene flow between seemingly disjunct *Maurolicus* populations and whether this is sufficient to prevent evolutionary divergence. Some populations may be in the process of allopatric speciation in different ocean basins. This may be the case with the ‘northern’ and ‘southern’ clades, between which no shared haplotypes were found, and we have therefore retained the separation of clades 4A and 4B in Fig. 6 until further data are available. Additional data, particularly a geographically widespread survey involving population-level genetic markers, are needed to verify the putative species boundaries identified here. Further studies of *Maurolicus* promise to provide valuable insights in the complex nature of speciation in the open ocean.

## Data Availability

Voucher specimens have been deposited into DJR’s private collection with additional material held in the collections of ZMUB. DNA sequences were submitted to Genbank with the following accession numbers: MT128722, MT132176-MT132329 and MT132767-MT132893.
